# Supplementary material for: Hierarchical Harmonization of Atom-Resolved Metabolic Reactions across Metabolic Databases
Source: Metabolites. 2021 Jun 30;11(7):431. doi: 10.3390/metabo11070431 (PMC8307411; doi:10.3390/metabo11070431)
Supplement: Supplementary file 1 [file metabolites-11-00431-s001.zip › metabolites-1266714-supplementary.pdf]

metabolites

MDPI

A

B

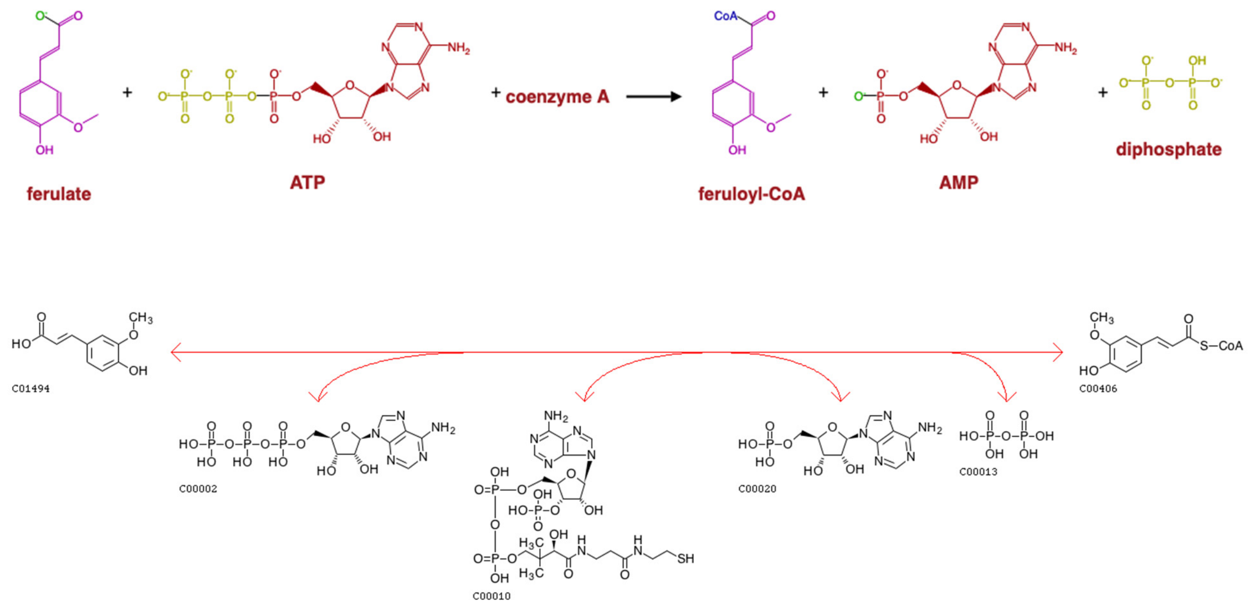

Figure S1. Reaction pair with mismatch of last EC number. A) MetaCyc reaction 6.2.1.34-RXN with EC number 6.2.1.34 (https://metacyc.org/META/NEW-IMAGE?object=6.2.1.34-RXN&&redirect=T); B) KEGG reaction R02194 with EC number 6.2.1.12 (https://www.genome.jp/entry/R02194).

A

B

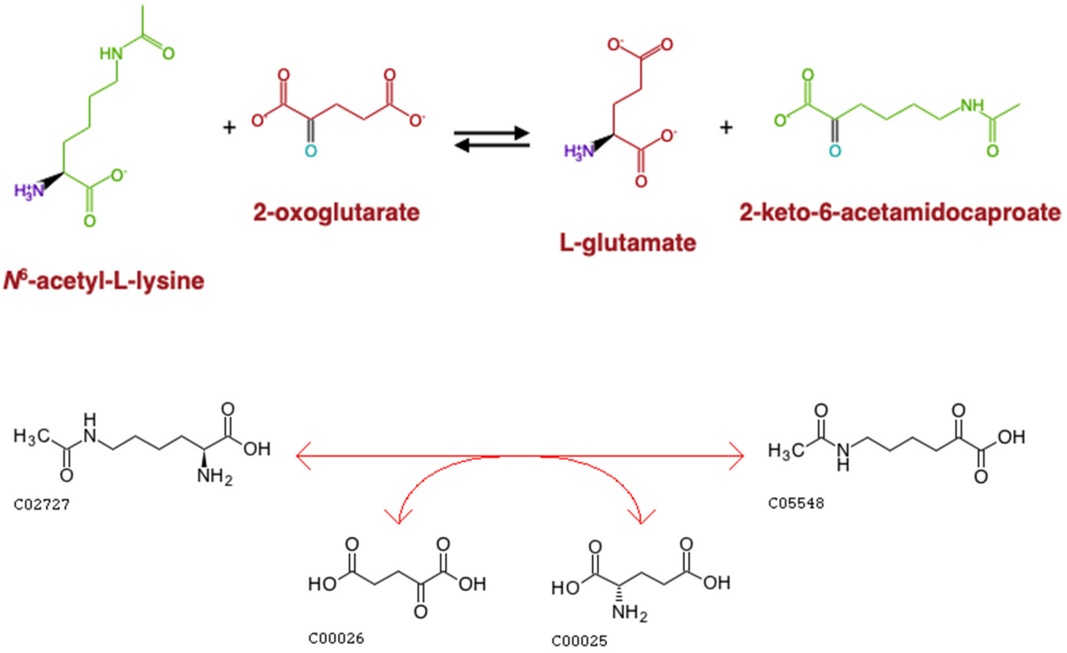

Figure S2. Reaction pair with missing 4th-level EC number designation. A) MetaCyc reaction ACETCAPR-RXN with EC number 2.6.1.- (https://metacyc.org/META/NEW-IMAGE?object=ACETCAPR-RXN&&redirect=T); B) KEGG reaction R04029 with EC number 2.6.1.65 (https://www.genome.jp/entry/R04029).

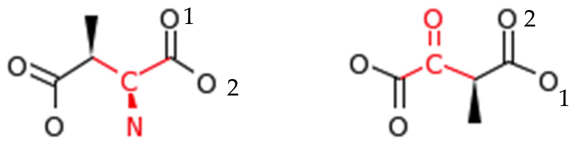

C03618 C06030

Figure S3. Example of atoms with interchangeable mappings. KEGG RPAIR maps atom 1 in C03618 to atom 2 in C06030 and atom 2 in C03618 to atom 1 in C06030.

A

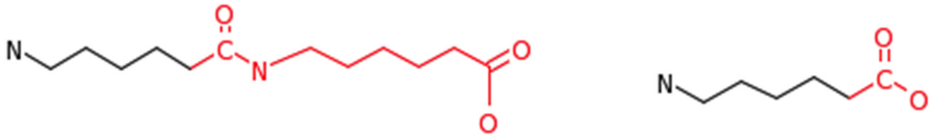

C01255 C02378

B

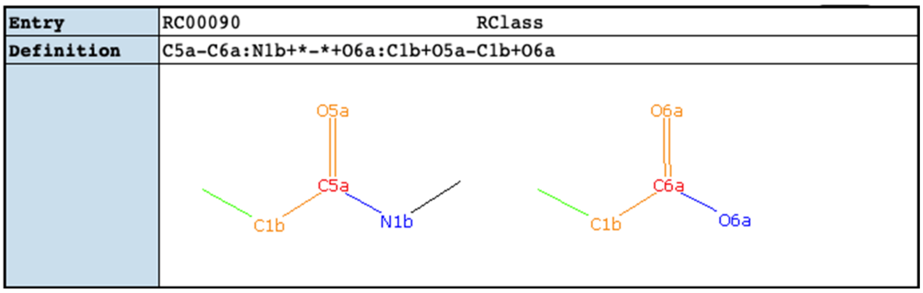

C

```
ENTRY         RP03127                       RPair
NAME          C01255_C02378
COMPOUND      C01255  N-(6-Aminohexanoyl)-6-aminohexanoate
              C02378  6-Aminohexanoate
TYPE          main
RDM           1
              1      N1b-N1a:C5a-*:C1b-C1b
RCLASS        RC00096
```

```
ALIGN         9
              1      1:N1b    9:N1a  #R1
              2      3:C1b    8:C1b  #M1
              3      6:C1b    5:C1b
              4      8:C1b    3:C1b
              5     10:C1b    1:C1b
              6     12:C1b    2:C1b
              7     14:C6a    4:C6a
              8     16:O6a    6:O6a
              9     17:O6a    7:O6a
              -      2:C5a      *    #D1
```

Figure S4. Comparison of KEGG RCLASS and RPAIR description for compound pair C01255 and C02378 (https://www.kegg.jp/kegg-bin/rpair_image?entry=RC00090&cpair=C01255_C02378). A) Compound C01255 and C02378; B) KEGG RCLASS description for the compound pair (https://www.genome.jp/dbget-bin/www_bget?rc:RC00090); C) KEGG RPAIR description for the compound pair along with the atom mappings.

Table S1. Hardly interpretable compound pairs.

RCLASS	Compound Pair
RC02715	C01054_C08626
RC00871	C01051_C02463
RC01850	C00751_C06309
RC01579	C00751_C06083
RC01851	C00751_C06310
RC01582	C01054_C01902
RC02163	C00751_C08627
RC02496	C12354_C18337
RC01862	C01054_C08615
RC01616	C05773_C05774
RC02632	C01054_C08637
RC03124	C01054_C08797
RC02708	C01054_C17966
RC01863	C01054_C08616
RC02603	C01054_C19819
RC01864	C01054_C08628
RC02714	C01054_C20188
RC02619	C01054_C19833
RC02620	C01054_C19801
RC02621	C00751_C19834
RC01901	C02094_C15943
RC02716	C01054_C20189
RC02717	C01054_C20191
RC02720	C01054_C20194
RC02722	C01054_C20200

Table S2. Harmonized reactions with inconsistent atom mappings.

MetaCyc	KEGG
1.13.11.45-RXN	['R05718']
1.14.11.18-RXN	['R05722']
12-ALPHA-L-FUCOSIDASE-RXN	['R04270']
2.1.1.21-RXN	['R01586']
2.3.1.56-RXN	['R04271']
2.3.1.90-RXN	['R00049']
2.4.1.121-RXN	['R03094']
2.4.1.230-RXN	['R07264', 'R11398']
2.4.1.54-RXN	['R07257']
2.5.1.41-RXN	['R04158']
2.5.1.42-RXN	['R04520']
2.5.1.67-RXN	['R08948']
2.6.1.50-RXN	['R02781']
2.6.1.80-RXN	['R07277']
2.7.1.121-RXN	['R01012']
2TRANSKETO-RXN	['R01067', 'R01830']
3.2.1.48-RXN	['R00801', 'R00802']
3.3.2.8-RXN	['R05784']
3.4.13.22-RXN	['R07651']
3.5.2.18-RXN	['R07984']
4-HYDROXYGLUTAMATE-AMINOTRANSFERASE-RXN	['R03266', 'R05052']
4-HYDROXYPHENYLPYRUVATE-DIOXYGENASE-RXN	['R02521']
4.1.3.26-RXN	['R08090']

4.2.1.100-RXN	['R05597']
4.2.3.10-RXN	['R02004']
4.2.3.19-RXN	['R05092']
4.2.3.20-RXN	['R02013', 'R06120']
4.3.1.16-RXN	['R00347', 'R05758', 'R09683']
4.3.1.20-RXN	['R00347', 'R05758', 'R09683']
4.6.1.11-RXN	['R02311', 'R09897']
6-PHOSPHO-BETA-GLUCOSIDASE-RXN	['R00838', 'R00839', 'R03256']
ACETYL-COA-ACETYLTRANSFER-RXN	['R00238']
ADENYL-KIN-RXN	['R00127']
ALDOSE-6-PHOSPHATE-REDUCTASE-NADPH-RXN	['R00834', 'R01817']
AMYGDALIN-BETA-GLUCOSIDASE-RXN	['R02985']
ARISTOLOCHENE-SYNTHASE-RXN	['R02307', 'R09574']
CATECHOL-OXIDASE-DIMERIZING-RXN	['R00080']
CONIFERIN-BETA-GLUCOSIDASE-RXN	['R02595']
DXPREDISOM-RXN	['R05688']
ETHANOLAMINE-PHOSPHATE-PHOSPHO-LYASE-RXN	['R00748']
F16ALDOLASE-RXN	['R01068', 'R01069', 'R01070']
FARNESYLTRANSTRANSFERASE-RXN	['R02061', 'R05555']
FORMALDEHYDE-TRANSKETOLASE-RXN	['R01440']
FPPSYN-RXN	['R02003', 'R08400', 'R08528']
GALPMUT-RXN	['R00505']
GERANYL-DIPHOSPHATE-CYCLASE-RXN	['R02007', 'R10509']
GLUCOSE-1-PHOSPHATE-PHOSPHODISMUTASE-RXN	['R00960']
GLUTAMATESYN-RXN	['R00114']
GLYCYRRHIZINATE-BETA-GLUCURONIDASE-RXN	['R03906']
HEMEOSYN-RXN	['R07411']
ISOCHORMAT-RXN	['R03037']
KETOLACTOSE-RXN	['R04783']
LACTOSE6P-HYDROXY-RXN	['R00838', 'R00839', 'R03256']
LANOSTEROL-SYNTHASE-RXN	['R03199']
MALTOSE-6-PHOSPHATE-GLUCOSIDASE-RXN	['R00838', 'R00839', 'R03256']
MALTOSE-SYNTHASE-RXN	['R00957']
METHYLMALONYL-COA-MUT-RXN	['R00833']
NITRATE-REDUCTASE-NADPH-RXN	['R00796']
O-AMINOPHENOL-OXIDASE-RXN	['R00074']
OHMETHYLBILANESYN-RXN	['R00084']
OLIGOGALACTURONIDE-LYASE-RXN	['R04382']
OXALOMALATE-LYASE-RXN	['R00477']
PENTALENENE-SYNTHASE-RXN	['R02305']
PHOSPHOENOLPYRUVATE-PHOSPHATASE-RXN	['R00208']
PROPIOIN-SYNTHASE-RXN	['R00038']
PRUNASIN-BETA-GLUCOSIDASE-RXN	['R02558']
RAUCAFFRICINE-BETA-GLUCOSIDASE-RXN	['R03703']
RXN-10005	['R09620']
RXN-10053	['R10049']
RXN-10482	['R09607', 'R10732']
RXN-10568	['R10734']
RXN-10600	['R09629']
RXN-10632	['R09123']
RXN-10635	['R09788']
RXN-10636	['R09526']
RXN-10637	['R09789']
RXN-10640	['R09630']
RXN-10685	['R08546']
RXN-10768	['R05759']

RXN-10775	['R09686']
RXN-11023	['R09249']
RXN-11141	['R10908']
RXN-11223	['R08807']
RXN-11224	['R08808']
RXN-11225	['R08809']
RXN-11226	['R08810']
RXN-11298	['R09588']
RXN-11485	['R09246']
RXN-11490	['R00039']
RXN-11501	['R03634']
RXN-11502	['R01103']
RXN-11747	['R07247']
RXN-11760	['R09533']
RXN-11767	['R00347', 'R05758', 'R09683']
RXN-11905	['R09615']
RXN-11910	['R09606']
RXN-12263	['R00702']
RXN-12326	['R09916']
RXN-12329	['R09689']
RXN-12338	['R09743']
RXN-12494	['R10734']
RXN-12573	['R10936']
RXN-12593	['R09903']
RXN-12625	['R00022']
RXN-12627	['R09942']
RXN-12774	['R08541', 'R09889', 'R10273']
RXN-12823	['R09888']
RXN-12824	['R09886', 'R09890']
RXN-12832	['R02311', 'R09897']
RXN-12836	['R09900']
RXN-12843	['R09908']
RXN-12846	['R09914']
RXN-12893	['R10079']
RXN-12983	['R06421', 'R09968']
RXN-13002	['R02007', 'R10509']
RXN-13074	['R10010']
RXN-13144	['R10050']
RXN-13218	['R08910']
RXN-13291	['R10194']
RXN-13335	['R10269']
RXN-13337	['R10009']
RXN-13338	['R10585']
RXN-13374	['R00032']
RXN-13642	['R10557', 'R10559']
RXN-13643	['R10558']
RXN-13724	['R02872']
RXN-13761	['R10271']
RXN-13762	['R10272']
RXN-13763	['R08541', 'R09889', 'R10273']
RXN-13769	['R10275']
RXN-14015	['R10284']
RXN-14092	['R10579']
RXN-14281	['R03802']
RXN-14282	['R05142']
RXN-14283	['R05141']

RXN-14573	['R10581']
RXN-14574	['R10582']
RXN-14633	['R10530']
RXN-14820	['R10527', 'R10539']
RXN-14821	['R10487', 'R10542']
RXN-14822	['R10527', 'R10539']
RXN-14823	['R10487', 'R10542']
RXN-14900	['R12095', 'R12250']
RXN-14901	['R12095']
RXN-14921	['R10623']
RXN-14922	['R10624']
RXN-14939	['R10583']
RXN-14940	['R10597']
RXN-14980	['R10580']
RXN-15224	['R08616']
RXN-15289	['R08127']
RXN-15419	['R10968']
RXN-15425	['R11087']
RXN-15605	['R10987']
RXN-15706	['R10897']
RXN-15708	['R10899']
RXN-15945	['R11040']
RXN-15946	['R10935']
RXN-15961	['R10380']
RXN-15964	['R10370']
RXN-17129	['R11410']
RXN-17138	['R11501']
RXN-17139	['R11503']
RXN-17175	['R11578']
RXN-17373	['R11327']
RXN-17501	['R07264', 'R11398']
RXN-17506	['R11544']
RXN-17772	['R11332']
RXN-1781	['R00015']
RXN-18377	['R11719']
RXN-18378	['R11718']
RXN-18379	['R11607']
RXN-18395	['R11705']
RXN-18396	['R09801']
RXN-18397	['R09800']
RXN-18398	['R11619']
RXN-18432	['R11593']
RXN-18496	['R11603']
RXN-18506	['R12114']
RXN-18592	['R11721', 'R11722']
RXN-18593	['R11721', 'R11722']
RXN-18659	['R11785']
RXN-18789	['R10004']
RXN-18791	['R08691']
RXN-18823	['R09788']
RXN-18824	['R09891']
RXN-18852	['R10004']
RXN-18854	['R09891']
RXN-18857	['R11942']
RXN-18975	['R11821']
RXN-18978	['R11853']

RXN-18979	['R11854']
RXN-18980	['R11855']
RXN-18999	['R11959']
RXN-19023	['R11862']
RXN-19024	['R11861']
RXN-19138	['R07543']
RXN-19150	['R07856']
RXN-19597	['R12087']
RXN-19768	['R10809']
RXN-20044	['R08691']
RXN-2543	['R07502']
RXN-2561	['R07503']
RXN-3962	['R00059']
RXN-4441	['R02727', 'R07265']
RXN-4781	['R02307', 'R09574']
RXN-4823	['R06523']
RXN-4882	['R09115']
RXN-5101	['R06421', 'R09968']
RXN-5106	['R05765', 'R09974']
RXN-5107	['R05766', 'R09975']
RXN-5109	['R09962']
RXN-5110	['R02009']
RXN-5121	['R09961']
RXN-5123	['R09962']
RXN-5142	['R02011', 'R09971', 'R09972']
RXN-5341	['R10040']
RXN-7929	['R00354']
RXN-8036	['R04998']
RXN-8046	['R07630']
RXN-8059	['R11626']
RXN-8147	['R05126']
RXN-8149	['R05127']
RXN-8151	['R03698']
RXN-8152	['R09402']
RXN-8414	['R08541', 'R09889', 'R10273']
RXN-8415	['R08373']
RXN-8416	['R10584']
RXN-8417	['R09614']
RXN-8418	['R10599']
RXN-8422	['R08695']
RXN-8424	['R10598']
RXN-8425	['R10284']
RXN-8426	['R09895']
RXN-8512	['R06303']
RXN-8540	['R08542']
RXN-8541	['R08540']
RXN-8546	['R09970']
RXN-8549	['R09619']
RXN-8551	['R10006']
RXN-8562	['R07648', 'R09558']
RXN-8563	['R05765', 'R09974']
RXN-8564	['R05766', 'R09975']
RXN-8565	['R09963']
RXN-8572	['R10726']
RXN-8574	['R08696']
RXN-8575	['R09963']

RXN-8576	['R02011', 'R09971', 'R09972']
RXN-8587	['R09886', 'R09890']
RXN-8588	['R09610', 'R09892', 'R10005']
RXN-8589	['R10004']
RXN-8591	['R09610', 'R09892', 'R10005']
RXN-8592	['R09610', 'R09892', 'R10005']
RXN-8593	['R09610', 'R09892', 'R10005']
RXN-8594	['R02311', 'R09897']
RXN-8599	['R10004']
RXN-8600	['R10004']
RXN-8601	['R10008']
RXN-8602	['R10006']
RXN-8603	['R10584']
RXN-8604	['R10007']
RXN-8608	['R08691']
RXN-8609	['R09886', 'R09890']
RXN-8621	['R09607', 'R10732']
RXN-8650	['R07597']
RXN-8651	['R07597']
RXN-8731	['R05166']
RXN-8801	['R10555']
RXN-8813	['R07475', 'R08748']
RXN-8931	['R08696']
RXN-8939	['R07648', 'R09558']
RXN-8958	['R09292']
RXN-8992	['R09248']
RXN-9030	['R11463']
RXN-9106	['R09249']
RXN-9138	['R06447']
RXN-9140	['R10937']
RXN-9349	['R07830']
RXN-9412	['R05784']
RXN-9413	['R05784']
RXN-9456	['R09714']
RXN-9457	['R09715']
RXN-9464	['R05784']
RXN-9588	['R10035']
RXN-9664	['R09913']
RXN-9674	['R10039']
RXN-9825	['R10452']
RXN-9850	['R04811']
RXN-9863	['R05419']
RXN-9962	['R09626']
RXN-9963	['R09627']
RXN0-3521	['R07661']
RXN0-4641	['R08555']
RXN0-5180	['R02061', 'R05555']
RXN0-5183	['R01444']
RXN0-5297	['R05134']
SERINE--PYRUVATE-AMINOTRANSFERASE-RXN	['R00585']
STERYL-BETA-GLUCOSIDASE-RXN	['R01460']
STRICTOSIDINE-BETA-GLUCOSIDASE-RXN	['R03820']
SUCROSE-PHOSPHORYLASE-RXN	['R00803']
TAGAALDOL-RXN	['R01068', 'R01069', 'R01070']
TECH1REDHAL-RXN	['R05402']
TRANS-HEXAPRENYLTRANSTRANSFERASE-RXN	['R09247']

TRANS-PENTAPRENYLTRANSFERASE-RXN	['R09245']
TRE6PHYDRO-RXN	['R00837']
TRICHODIENE-SYNTHASE-RXN	['R02306']
TRYPSYN-RXN	['R02722']
TYROSINE-3-MONOOXYGENASE-RXN	['R07212']
UDPNACETYLGLUCOSAMENOLPYRTRANS-RXN	['R00660']
URATE-RIBONUCLEOTIDE-PHOSPHORYLASE-RXN	['R02646']
UROGENIIISYN-RXN	['R03165']
VICIANIN-BETA-GLUCOSIDASE-RXN	['R03642']
X-METHYL-HIS-DIPEPTIDASE-RXN	['R03288']
